# The perils of COVID-19 in Nepal: Implications for population health and nutritional status

**DOI:** 10.7189/jogh.10.010378

**Published:** 2020-06

**Authors:** Devendra Raj Singh, Dev Ram Sunuwar, Bipin Adhikari, Sylvia Szabo, Sabu S Padmadas

**Affiliations:** 1 Department of Public Health, Asian College for Advance Studies, Purbanchal University, Lalitpur, Nepal; 2 Department of Nutrition and Dietetics, Nepal APF Hospital, Kathmandu, Nepal; 3 Nuffield Department of Clinical Medicine, University of Oxford, Oxford, UK; 4 Department of Development and Sustainability, Asian Institute of Technology, Pathum Thani, Thailand; 5 Department of Social Statistics and Demography, University of Southampton, Southampton, UK

COVID-19, an infectious disease from a highly contagious coronavirus (SARS-CoV-2), continues to spread rapidly around the globe since its outbreak as an unknown respiratory infections from Wuhan city of China in December 2019 [1]. As of May 31, 2020, globally there were more than 6.1 million cases of COVID-19 confirmed with a death toll surpassing 371 000. In Nepal, over 1400 cases of COVID-19 were confirmed with six deaths reported as of May 31, 2020 [2]. In the absence of an effective antiviral or immunomodulatory treatment for COVID-19, current pandemic control and management rely heavily on public health interventions such as social distancing and locking down the cities to prevent further spread [3].

By the end of March 2020 more than 100 countries have decided to implement either partial or full lockdown, thus compelling millions of people to stay at home [4]. Nepal implemented full lockdown since March 24, 2020 initially for 12 days [5], and since then extended for an additional four week period and further until June 14, closely following the epidemiological patterns in and outside Nepal, particularly neighboring India. Although Nepal has already closed down its borders with India and China, transnational migration of workers returning from India and those stranded at the border elevates the risk of disease transmission. There is a growing concern that the long-term extension of lockdown strategy can severely affect the health and nutrition security of the poor and vulnerable population in Nepal.

**Figure 1:**
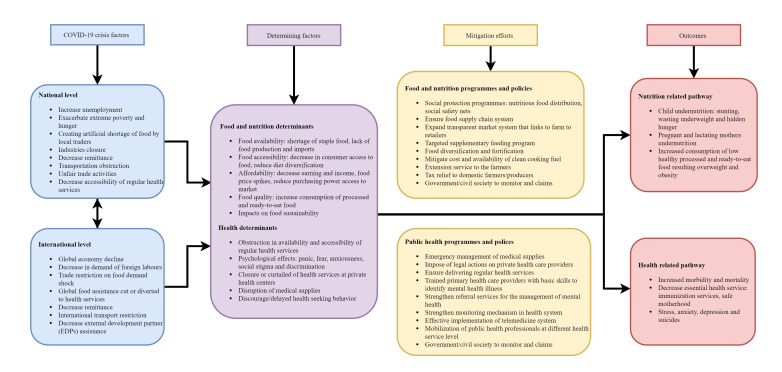
Pathways showing COVID-19 perils on public health and nutritional outcomes in Nepal.

Nepal continues to be vulnerable to extreme poverty, severe malnutrition, and infections. The country is yet to recover from the adverse impact of the devastating aftermath of the 2015 earthquakes [6]. The current lockdown has already exacerbated the health risks and health care resources, and are likely to continue beyond the COVID-19 pandemic. Even after the exit from the lockdown, the current global public health crisis will have a long-lasting cataclysmic impact on the lives of the people and societies at national and global levels (**Figure 1**). The framework provides an illustration of the underlying factors and the potential mitigating interventions for health and nutrition outcomes. We examine how COVID-19 lockdown impacts public health systems and nutritional outcomes in Nepal.

First, Nepal has a high burden of maternal mortality (239 maternal deaths per 100 000 live births) and child mortality (39 deaths per 1000 live births) [7]. These indicators are likely to worsen following the lockdown measures due to challenges in accessing emergency and regular health services. The major public health challenge for Nepal is the lack of access to health care against the backdrop of weak and inadequate public health infrastructure [8]. Despite the government’s efforts to set up hospitals for treating COVID-19 patients, the majority of private health care services have halted their services. This has had a negative impact on health care, severely affecting reproductive, maternal, and child health services and those with chronic health conditions and non-communicable diseases. A recent report from the government source confirmed the death of a 22 year-old female who had postpartum hemorrhage following a home birth but could not seek timely care as the nearest health facility was closed [9]. Due to COVID-19 and the lockdown, an increasing number of women are giving birth at home, where unsafe delivery methods and unhygienic conditions continue to elevate infection risks and maternal complications, while others are at risk of dying at home due to lack of ambulance and emergency care services [9].

A few patients with co-morbid conditions developing infection symptoms have lost their lives because of tepid response from private hospitals such as declined admissions, negligence, and unnecessary referrals, apparently due to fear of COVID-19. Also, routine immunization services have been suspended. Approximately, three million Nepalese children aged nine months to five years have missed their regular vaccination schedules. During this lockdown, an outbreak of measles with 150 cases and two deaths have been reported from a rural region in Dhading district [9]. As health systems continue to combat COVID-19, Nepal is at high risk of facing detrimental effects on (public) health care, further constraining the health resources of the country.

Second, prolonged exposure to hunger and malnutrition can trigger infections, cognitive-developmental deficit (in young children), behavioral and mental dysfunctions such as stress, suicides, and depression in both minors and adults; and can aggravate chronic conditions such as asthma, obesity, hypertension, diabetes, and hyperlipidemia [10,11]. The consequences of the COVID -19 crisis on mental health are profound among Nepalese who are already struggling with the adverse impact of poverty, hunger, and natural disasters. Due to the loss of wages, the most vulnerable elderly and children living in poor households are left with no choices but skip their meals and risk chronic health conditions [11].

**Figure:**
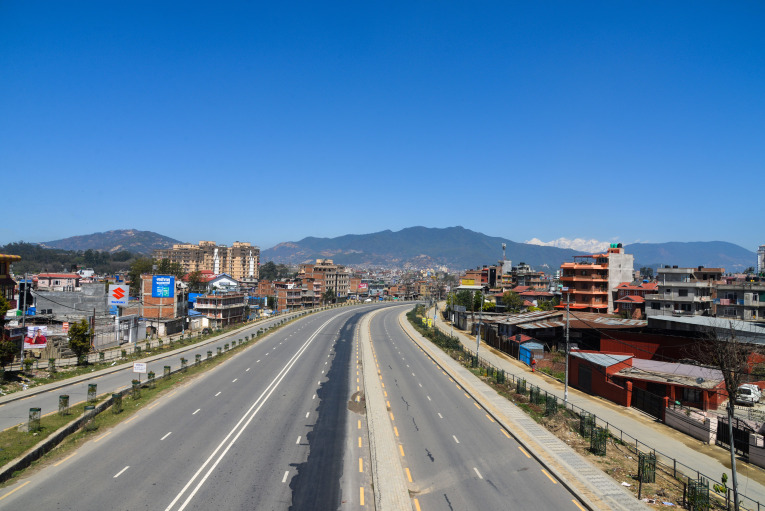
Photo: Empty streets of Kathmandu city due to nationwide complete lockdown, the capital of Nepal (used from the collection of Pramod Bhattarai photo gallery with permission).

The government has limited capacity and expertise to chart any emergency measures to address these pertinent health issues, as the available resources are fully channeled towards combatting COVID-19. Prolonged exposure to malnutrition can weaken the immune system, especially during an epidemic outbreak. In Nepal, the coexistence of the double burden of malnutrition aggravates COVID-19 exposure and can perpetuate the vicious cycle of weakened immunity and infection [12]. Historically, Nepal has been facing food and nutrition security challenges with the highest prevalence of double and triple burden of malnutrition [13]. The prevalence of stunting, underweight, wasting among under-five children, and anemia among women of reproductive age are already among the highest in Nepal when compared to other South Asian countries [7]. The COVID-19 outbreak may exacerbate the extensive losses related to food production and food supply chain, thus putting the migrant and labor population at risk of hunger and malnutrition.

The COVID-19 pandemic is evolving at different stages across the countries, and it is difficult to predict when it will be over. A recent report suggested that approximately an additional 130 million people will face starvation due to the COVID-19 crisis [12]. National and international stakeholders and government authorities should take urgent actions to ensure food and nutrition security to the population. It is crucial to ensure the availability, accessibility, and affordability of a healthy and nutritious diet in the food system. For example, during the Ebola outbreak in Guinea, the humanitarian community together with the government ensured household food security, by providing super cereal+ and other commodities including rice, lentils, peas, beans, oil salt, and sugar [14]. Ready-to-use therapeutic foods (RUTF) for severe acute malnutrition and replacement milk for children who could not be breastfed were also provided [14]. In addition, strict monitoring and regulating food prices are critical. E-commerce and online home delivery systems can be an effective alternative. In rural regions, however, subsistence farming can be encouraged and supported to prevent food shortages.

Nepal suffers from an unequal distribution of health care services in hard-to-reach rural areas, especially in hill and mountainous regions [15]. Most private health care institutions in Nepal are urban-centric, and seem reluctant to show any empathy and commitment to people during the current crisis. We urge the government of Nepal to enforce legislation to ensure accountability and responsibility of private health care sectors in responding to the current health crisis, and fast-tracking provision of essential medical supplies and human resources. Equally important, health systems should ensure critical care interventions to screen, diagnose, and treat COVID-19 patients across the country. As the uncertainty associated with COVID-19 looms large, an effective health intervention would be to train and devolve responsibility to primary health care providers to provide treatment, referral, follow-up, and mental health counseling, targeting those at high risk including pregnant women, children and elderly. The government of Nepal should take urgent steps to continue routine immunization programs and maintain essential maternal and child health care services.
